# Calprotectin and TNF trough serum levels identify power Doppler ultrasound synovitis in rheumatoid arthritis and psoriatic arthritis patients in remission or with low disease activity

**DOI:** 10.1186/s13075-016-1032-z

**Published:** 2016-07-08

**Authors:** José Inciarte-Mundo, Julio Ramirez, Maria Victoria Hernández, Virginia Ruiz-Esquide, Andrea Cuervo, Sonia Raquel Cabrera-Villalba, Mariona Pascal, Jordi Yagüe, Juan D. Cañete, Raimon Sanmarti

**Affiliations:** Department of Rheumatology, Hospital Clinic, University of Barcelona, Carrer Villarroel 170, 08036 Barcelona, Spain; Department of Immunology, Hospital Clinic, University of Barcelona, Barcelona, Spain

**Keywords:** Calprotectin, TNFi trough serum levels, Ultrasound, Disease activity, Rheumatoid arthritis, Psoriatic arthritis

## Abstract

**Background:**

Serum levels of calprotectin, a major S100 leucocyte protein, are associated with disease activity in rheumatoid arthritis (RA) and psoriatic arthritis (PsA) patients. Higher drug trough serum levels are associated with good response in patients treated with tumour necrosis factor inhibitors (TNFi). Power Doppler ultrasound (PDUS) synovitis is predictive of flare and progression of structural damage in patients in clinical remission. The purpose of this study was to analyse the accuracy of calprotectin and TNFi trough serum levels in detecting PDUS synovitis in RA and PsA patients in clinical remission or with low disease activity who were receiving TNFi.

**Methods:**

We conducted a cross-sectional study of 92 patients (42 with RA, 50 with PsA) receiving adalimumab (ADA), etanercept (ETN) or infliximab who were in remission or had low disease activity (28-joint Disease Activity Score based on erythrocyte sedimentation rate <3.2). Associations of calprotectin, TNFi trough serum levels and acute phase reactants with PDUS synovitis were assessed using correlation and linear regression analyses. The accuracy and discriminatory capacity in detecting PDUS synovitis was assessed using ROC curves.

**Results:**

PDUS synovitis was found in 62.4 % of RA patients and 32 % of PsA patients. Both RA and PsA patients with PDUS synovitis had higher calprotectin levels and lower TNFi trough serum levels. Calprotectin positively correlated with ultrasound scores (all *r* coefficients >0.50 in RA). Calprotectin correlated with the PDUS synovitis score in patients treated with ADA and ETN. Using PDUS synovitis (yes or no) as the reference variable, calprotectin had an AUC of 0.826. The best cut-off was ≥1.66 μg/ml, with a likelihood ratio of 2.77. C-reactive protein (AUC 0.673) and erythrocyte sedimentation rate (AUC 0.731) had a lower discriminatory capacity. TNFi trough serum levels were significantly associated with PDUS synovitis (OR 0.67, 95 % CI 0.52–0.85, *p* < 0.001) but their accuracy (AUC <0.5) was less than that of calprotectin. TNFi trough serum levels were inversely correlated with calprotectin and PDUS synovitis in RA and PsA patients receiving ADA and ETN.

**Conclusions:**

Calprotectin and TNFi trough serum levels may help identify PDUS synovitis in RA and PsA patients in clinical remission or with low disease activity.

## Background

New therapeutic strategies, including early therapy, treat-to-target approaches and biological therapies, have led to substantial improvements in the prognosis of patients with rheumatoid arthritis (RA) and other types of inflammatory arthritis, such as ankylosing spondylitis and psoriatic arthritis (PsA) [1]. These strategies have enabled patients to reach clinical remission (i.e., the absence of clinically detectable joint inflammation), which is now a realistic therapeutic goal in clinical practice [2]. However, there is no consensus on what constitutes clinical remission and which indices should be used to measure it, and therefore rates of clinical remission depend on the articular index used. Furthermore, most patients in clinical remission have evidence of inflammatory activity, especially when less stringent criteria are used [3–6].

Musculoskeletal ultrasound (MSUS), a non-invasive diagnostic technique, is widely used in rheumatology to measure joint inflammation with greater sensitivity [7]. Power Doppler (PD) detects synovial flow, a sign of increased synovial vascularization, and better reflects active synovial inflammation compared with synovial hypertrophy (SH) [8]. PD correlates with disease activity and decreases after treatment, and it is therefore sensitive to change [9–11]. MSUS has revealed that a significant proportion of patients classified as being in clinical remission exhibit power Doppler ultrasound (PDUS) synovitis, which may explain the joint damage progression observed in some patients despite no clinical findings of disease activity. In addition, PDUS synovitis is predictive of clinical flares [12–15].

In rheumatology, promising biomarkers that more accurately reflect the clinical status, including remission, of patients with inflammatory arthritis have been recognized. Use of these biomarkers might enhance clinical practice and guide therapeutic decisions. Recently, serum calprotectin, a heterodimeric complex of two S100 calcium-binding myeloid-related proteins (MRP8 [or S100A8] and MRP14 [or S100A9]), has been reported to be a sensitive biomarker of disease activity in patients with RA and patients with spondyloarthritis [16]. Calprotectin is an important proinflammatory factor of innate immunity, acting as endogenous damage-associated molecular pattern molecules via Toll-like receptor 4 activation [17]. Higher calprotectin levels have been found in the synovial fluid and serum of RA and PsA patients [18]. Serum calprotectin levels correlate with disease activity and are independently associated with radiographic progression in RA [19–21]. Levels decrease significantly after treatment with tumour necrosis factor inhibitors (TNFi) and might be useful in monitoring biological therapy [22, 23].

Recent studies have found a significant dose-response relationship between the extent of clinical improvement and TNFi trough serum levels [24, 25], with higher drug trough serum levels being associated with good responses due to significant decreases in disease activity [26], less progression of joint damage [27] and reductions in acute phase response proteins such as C-reactive protein (CRP) [28].

The aim of this study was to analyse the accuracy of calprotectin serum levels and TNFi trough serum levels in detecting PDUS synovitis in RA and PsA patients in clinical remission or with low disease activity who were receiving TNFi.

## Methods

This cross-sectional study included consecutive RA (American College of Rheumatology 1987 criteria) and polyarticular PsA (CASPAR, ClASsification criteria for Psoriatic Arthritis) patients from our arthritis unit. All patients were in remission (28-joint Disease Activity Score based on erythrocyte sedimentation rate [DAS28-ESR] ≤2.6) or had low disease activity (DAS28-ESR ≤3.2) in two consecutive visits ≥3 months apart and had been taking adalimumab (ADA), etanercept (ETN) or infliximab (IFX) for ≥3 months. Patients with DAS28-ESR >3.2 or PsA patients with axial or entheseal involvement or an oligoarticular peripheral pattern were excluded. Information collected comprised demographic data, disease duration, autoantibody status, radiological data, concomitant conventional synthetic disease-modifying anti-rheumatic drug (csDMARD) therapy, and dose and duration of biological therapy. Some patients were receiving reduced-dose biological therapy due to persistent remission and/or low disease activity. A reduced dose was defined as treatment with a Lower amount of the drug or longer interval of administration than recommended in each product package insert.

### Measurement of disease activity

Before MSUS assessment, all patients underwent a clinical assessment including 28-joint swollen and tender joint counts as well as physician and patient global assessments with visual analogue scales (0–100 mm). Three composite disease activity indices were subsequently calculated: DAS28-ESR [29, 30], Simple Disease Activity Index [31] and Clinical Disease Activity Index [32].

### Ultrasound assessment

Sonographic assessments were carried out using high-sensitivity ultrasound equipment (MyLab Twice®; Esaote, Genoa, Italy), with a frequency range from 10 to 14 MHz and a pulse repetition frequency between 500 and 800 Hz. Receiver gain settings were controlled to eliminate artefacts. Joint MSUS findings were defined according to published Outcome Measures in Rheumatoid Arthritis Clinical Trials (OMERACT) definitions [33]. A single experienced sonographer (JR) who was blinded to the results of the clinical joint examination evaluated 11 joints and tendons of each hand (including proximal interphalangeal joints, metacarpophalangeal joints, and wrists) for both SH and intra-articular PD signalling according to European League Against Rheumatism guidelines [34]. SH and PD signals were graded using a four-grade semi-quantitative scoring system (0 = no, 1 = mild, 2 = moderate and 3 = severe) according the methodology of Szkudlarek et al [35]. Intra-rater agreement on MSUS assessment, calculated as previously described [36], were 0.83 for SH and 0.90 for PD.

By summing the scores for elementary lesions in each joint, we calculated the PD score (sum of PD scores in all joints, range 0–66), the SH score (sum of SH scores in all joints, range 0–66) and the global score (sum of the PD and SH scores, range 0–132). PDUS synovitis was defined as a PD signal in synovial tissue [37]. A more stringent definition of active synovitis (ultrasound-defined active synovitis [UdAS]) developed by our group (SH grade ≥2 plus PDUS synovitis signal) was also recorded. UdAS patients showed significantly higher serum levels of several angiogenic factors thought to be relevant to RA pathogenesis. Clinically, UdAS-positive patients showed greater disease activity on the basis of composite indices different from those used for UdAS-negative patients [36].

### Measurement of serum calprotectin levels, acute phase response and TNFi trough serum levels

Blood samples were collected immediately before the next administration of TNFi, centrifuged 20 minutes after sample collection to separate serum and plasma, and stored at −80 °C until analysis. Calprotectin serum levels were determined using an enzyme-linked immunosorbent assay (ELISA) test kit (CALPROLAB Calprotectin ELISA (ALP); Calpro AS, Lysaker, Norway) in accordance with the manufacturer’s protocol. Briefly, 100 ml of each standard, control and diluted 1:20 sample in duplicate wells were incubated at room temperature for 40 minutes. Three washings were done, 100 ml of the conjugated enzyme were added, and the plates were incubated at room temperature for 40 minutes. After three washes and the addition of the enzyme substrate, the optical density values at 405 nm were determined using an ELISA reader. To reduce variation in calprotectin determination, the whole procedure was performed in a Triturus® autoanalyzer (Diagnostic Grifols, Barcelona, Spain) [38]. ESR was measured using the Westergren method (Normal Value (NV) <20 mm/h), and CRP was measured by nephelometry (lowest detection limit 0.01 mg/dl, NV <0.8 mg/dl). Trough serum levels of ETN, ADA and IFX were measured by using a bridging ELISA (Promonitor®; Progenika Biopharma, Derio, Spain) that has been validated and shows adequate correlation in measuring drug levels and anti-drug antibodies [39].

The study was conducted in accordance with the Declaration of Helsinki and was approved by the Hospital Clinic of Barcelona Clinical Research Ethics Committee (Reg. 2013/8382). Signed informed consent was obtained from all patients before study enrolment.

### Statistical analysis

Continuous data were presented as medians and ranges, and categorical variables were presented as absolute frequencies and percentages. Groups were compared using Student’s *t* test or the Mann-Whitney *U* test when appropriate. Correlations were assessed using Spearman’s correlation coefficient. Logistic regression models were used to assess associations between calprotectin, TNFi trough serum levels and PDUS synovitis, using ultrasound (US) as the dependent variable and calprotectin and TNFi trough serum levels as independent factors. Crude ORs with 95 % CIs were calculated. Multivariate models were constructed to analyse the effect of covariates and to fully adjust the association between calprotectin, TNFi trough serum levels and PDUS synovitis. Models were fitted separately and compared using the Akaike information criterion and the Bayesian information criterion. The discriminatory capacity of calprotectin, TNFi trough serum levels, CRP and ESR, with PDUS synovitis (yes or no) as the gold standard, was analysed using ROC curves, and the best cut-off in terms of sensitivity and specificity was identified. The predictive values, accuracy and positive likelihood ratio were calculated. The AUC was estimated using Hanley’s corrected confidence intervals. The analyses were carried out using STATA version 11 software (StataCorp, College Station, TX, USA).

## Results

Ninety-two patients were included (42 RA, 50 PsA), and their median disease duration was 15 (1–44) years. Forty-four patients were receiving ETN (22 RA and 22 PsA), 32 were taking ADA (14 RA and 18 PsA) and 16 were receiving IFX (6 RA and 10 PsA). The median biological treatment duration was 63.4 (12–166) months, and 42 patients had received a reduced dose of biological therapy. Seventy-one patients (77.2 %) were in remission, and 21 (22.8 %) had low disease activity. PsA patients included were younger, had a shorter duration of biological therapy, and had lower percentages of csDMARD and steroid use than RA patients (Table 1).

**Table 1:** Patients and disease characteristics

Patient characteristics	Total *(n* = 92)	RA (*n* = 42)	PsA (*n* = 50)	*p* Value
Female sex, *n* (%)	59 (64.1)	34 (81)	25 (50)	0.158
Age, years, median (range)	58 (30–81)	63.5 (30–81)	54.5 (33–77)	<0.001
Body index mass, kg/m^2^, median (range)	26.4 (18–42)	26.2 (19.2–42)	26.6 (18.3–35)	0.189
Disease duration, years, median (range)	15 (1–44)	15.5 (2–44)	14.5 (1–36)	0.785
Presence of erosions, *n* (%)	53(57.6)	33 (78.6)	20 (40)	0.012
At least one previous biological treatment, *n* (%)	28 (27)	11 (26)	14 (28)	0.552
Concomitant csDMARD, *n* (%)	47 (51.1)	32 (76.2)	15 (30)	0.005
Concomitant steroids, *n* (%)	15 (16.3)	13 (31)	2 (4)	<0.001
Prednisone dose, mg/day, median (range)	2.5 (3–5)	2.5 (3–5)	3.7 (3–5)	0.152
Biological treatment duration, months, median (range)	64.8 (12–166)	83.2 (9–165)	58.3 (7.6–166)	0.017
Reduced dosage,^a^ *n* (%)	42 (45.7)	12 (28.6)	30 (60)	<0.001
Albumin, g/dl, median (range)	42.5 (30–49)	32 (30–49)	47 (30–49)	0.005
CRP, mg/dl, median (range)	0.095 (0.01–1.45)	0.10 (0.01–1.4)	0.09 (0.01–0.6)	0.288
ESR, mm/h, median (range)	10 (2–43)	12.5 (2–43)	8.5 (2–32)	0.004
Calprotectin, μg/ml, median (range)	1.67 (0.06–5.54)	2.16 (0.2–5.5)	1.36 (0.06–4.6)	0.002
SJC, median (range)	0 (0–3)	0 (0–3)	0 (0–2)	0.625
TJC, median (range)	0 (0–2)	0 (0–2)	0 (0–1)	0.788
DAS28-ESR, median (range)	1.96 (1.0–3.2)	2.31 (1.3–3.2)	1.82 (1–3.1)	<0.001
Remission based on DAS28-ESR, *n* (%)	71 (77.2)	27 (64.3)	44 (88)	0.005
Low disease activity based on DAS28-ESR, *n* (%)	21 (22.8)	15 (35.7)	6 (12)	0.005
CDAI, median (range)	6 (2–11.0)	6 (2–11)	6 (2–8)	0.782
SDAI, median (range)	6 (2–11.1)	6 (2–11)	6 (2–8)	0.005

*Abbreviations: CDAI* Clinical Disease Activity Index, *CRP* C-reactive protein, *csDMARD* conventional synthetic disease-modifying anti-rheumatic drug, *DAS28-ESR* 28-joint Disease Activity Score based on erythrocyte sedimentation rate, *ESR* erythrocyte sedimentation rate, *PsA* psoriatic arthritis, *RA* rheumatoid arthritis, *SDAI* Simple Disease Activity Index, *SJC* swollen joint count, *TJC* tender joint count

^a^Treatment regimen with a lesser amount of the drug or longer interval of administration than those recommended in the package insert for each product

### Power Doppler ultrasound synovitis and disease status

Forty-three patients (46.7 %) had PDUS synovitis (27 RA [64.2 %] and 16 PsA [32 %]), of whom 15 (10 RA [23.8 %] and 5 PsA [10 %]) met the criteria for UdAS (SH grade ≥2 plus PDUS synovitis signal). Patients with PDUS synovitis were mostly female, had a diagnosis of RA, were more frequently treated with steroids, and had a higher percentages of low disease activity according to all indices assessed (Table 2). Similar results were obtained when UdAS criteria were applied (data not shown).

**Table 2:** Calprotectin, disease activity, ultrasound assessment and tumour necrosis factor inhibitor trough serum levels according to diagnosis

Patient characteristics	RA (*n* = 42)		PsA (*n* = 50)	
	PDUS-negative (*n* = 15)	PDUS-positive (*n* = 27)	PDUS-negative (*n* = 34)	PDUS-positive (*n* = 16)
Female sex, *n* (%)	11 (73.3)	23 (85.2)	15 (44.1)	10 (62.5)^a^
Age, years, median (range)	62 (49–78)	64 (30–81)	53 (33–77)	55 (40–72)
Disease duration, years, median (range)	13 (8–28)	17 (2–44)	13.5 (1–28)	16 (3–36)
Concomitant csDMARD, *n* (%)	11 (73.3)	21 (77.8)	11 (32.4)	4 (25)
Reduced dose, *n* (%)	4 (26.7)	8 (29.6)	21 (61.8)	9 (56.3)
Calprotectin, μg/ml, median (range)	1.44 (0.2–2.4)	2.95 (0.2–5.5)^a^	0.70 (0.06–3.7)	2.36 (0.9–4.6)^b^
CRP, mg/dl, median (range)	0.07 (0.02–0.1)	0.30 (0.01–1.4)^a^	0.08 (0.01–0.6)	0.09 (0.01–0.3)
ESR, mm/h, median (range)	10 (2–24)	13 (2–43)	8 (2–29)	13 (4–32)^a^
SJC, median (range)	0 (0–1)	0 (0–3)	0 (0–1)	0 (0)
TJC, median (range)	0 (0–1)	0 (0–2)	0 (0–1)	0 (0–1)
DAS28-ESR, median (range)	2.08 (1.5–2.6)	2.62 (1.3–3.2)^a^	1.67 (1–2.7)	2.15 (1.1–3.1)^a^
Remission based on DAS28-ESR, *n* (%)	13 (86.7)	14 (51.9)^b^	9 (90)	6 (75)^a^
Low disease activity based on DAS28-ESR, *n* (%)	2 (13.3)	13 (48.1)^b^	1 (10)	2 (25)
SDAI, median (range)	6.02 (2–8)	6.26 (2–11)^a^	5.10 (2–8)	6.04 (2–8.3)
CDAI, median (range)	6 (2–8)	6 (2–11)	5 (2–8)	6 (2–8)
ADA trough serum levels, μg/ml, median (range)	8.39 (4.2–12)	1.68 (0.6–12)^a^	6.95 (4.1–12)	0.88 (0.2–9.8)^a^
ETN trough serum levels, μg/ml, median (range)	2.54 (0.2–4.7)	0.98 (0.7–2.3)^a^	1.38 (01–3.5)	0.91 (0.6–1.6)^a^
IFX trough serum levels, μg/ml, median (range)	8.39 (4.2–12)	1.68 (0.6–12)^a^	3.21 (0.7–7.7)	2.86 (0.1–6.5)

*Abbreviations: ADA* adalimumab, *CDAI* Clinical Disease Activity Index, *CRP* C-reactive protein, *csDMARD* conventional synthetic disease-modifying anti-rheumatic drug, *DAS28-ESR* 28-joint Disease Activity Score based on erythrocyte sedimentation rate, *ESR* erythrocyte sedimentation rate, *ETN* etanercept, *IFX* infliximab, *PDUS* power Doppler ultrasound, *PsA* psoriatic arthritis, *RA* rheumatoid arthritis, *SDAI* Simple Disease Activity Index, *SJC* swollen joint count, *TJC* tender joint count

^a^ *p* < 0.05

^b^ *p* < 0.001

### Calprotectin, acute phase reactants and power Doppler ultrasound synovitis

Patients with PDUS synovitis had higher levels of calprotectin (PDUS-negative [*n* = 49] 1 μg/ml [0.6–3.7] vs. PDUS-positive [*n* = 43] 2.68 μg/ml [0.22–5.5], *p* < 0.001), CRP (PDUS-negative [*n* = 49] 0.07 mg/dl [0.1–0.6] vs. PDUS-positive [*n* = 43] 0.20 mg/dl [0.01–1.4], *p* = 0.005) and ESR (PDUS-negative [*n* = 49] 8 mm/h [2–29] vs. PDUS-positive [*n* = 43] 13 mm/h [2–43], *p* < 0.001). Similar results were obtained when patients were analysed according to diagnosis (RA vs. PsA), although no significant differences in CRP levels were observed between PsA patients with versus without PDUS synovitis (Table 2).

When a more stringent active synovitis criterion (UdAS) was applied, calprotectin (UdAS [*n* = 15] 3.48 μg/ml [0.2–5.5] vs. non-UdAS [*n* = 77] 1.47 μg/ml [0.06–4.8], *p* < 0.001), but not CRP (UdAS [*n* = 15] 0.11 mg/dl [0.05–0.24] vs. non-UdAS [*n* = 77] 0.09 mg/dl [0.07–0.13], *p* = 0.699) or ESR (UdAS [*n* = 15] 12.8 mm/h [8.1–20.1] vs. non-UdAS [*n* = 77] 9.6 mm/h [8.3–10.9], *p* = 0.108) distinguished patients with UdAS from those without UdAS.

Calprotectin strongly correlated with MSUS scores (all *r* coefficients >0.50 in RA patients). A weak correlation was found between ESR and joint indices and MSUS scores, while CRP correlated only with the PD score in RA (Table 3). Calprotectin correlated with the PD score in patients treated with ADA (ρ = −0.591, *p* < 0.001) and ETN (ρ = −0.313, *p* = 0.039), but not in those treated with IFX.

**Table 3:** Correlation between musculoskeletal ultrasound scores and calprotectin, acute phase reactants and disease activity indices

	RA (*n* = 42)	PsA (*n* = 50)
Doppler score		
Calprotectin	0.570^a^	0.420^b^
CRP	0.338^b^	0.058
ESR	0.285	0.337^b^
Albumin	−0.259	−0.322^b^
DAS28-ESR	0.380^b^	0.384^b^
SDAI	0.126	0.071
CDAI	0.080	0.115
SH score		
Calprotectin	0.589^a^	0.423^b^
CRP	0.044	0.092
ESR	0.217	0.020
Albumin	−0.336^b^	−0.210^b^
DAS28-ESR	0.423^b^	0.284^b^
SDAI	0.275	0.217
CDAI	0.163	0.277
Global score		
Calprotectin	0.641^a^	0.446^a^
CRP	0.105	0.122
ESR	0.226	0.111
Albumin	−0.332^b^	−0.237
DAS28-ESR	0.446^b^	0.348^b^
SDAI	0.280	0.195
CDAI	0.172	0.266

*Abbreviations: CDAI* Clinical Disease Activity Index, *CRP* C-reactive protein, *DAS28-ESR* 28-joint Disease Activity Score based on erythrocyte sedimentation rate, *ESR* erythrocyte sedimentation rate, *PsA* psoriatic arthritis, *RA* rheumatoid arthritis, *SDAI* Simple Disease Activity Index, *SH* synovial hypertrophy

Data presented are Spearman’s ρ correlations

^a^ *p* < 0.001

^b^ *p* < 0.05

**Fig. 1:**
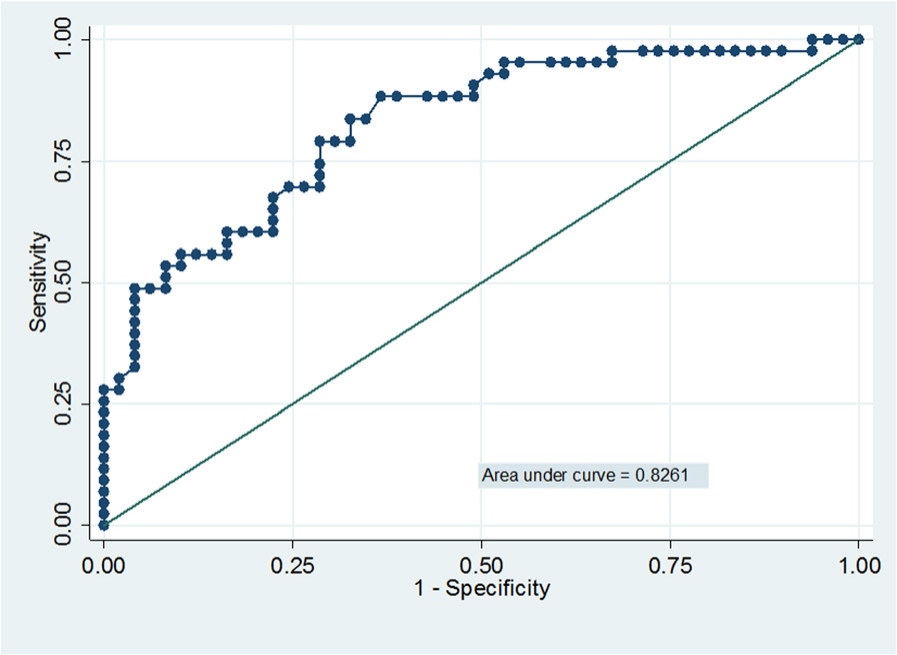
ROC curve fitting calprotectin discriminatory capacity to detect power Doppler ultrasound synovitis

The accuracy analysis with PDUS synovitis (yes or no) as the reference variable showed an AUC of 0.826 (95 % CI 0.742–0.910) and a cut-off calprotectin level of 1.66 μg/ml (sensitivity 79.1 %, specificity 83.4 %). The positive likelihood ratio of this calprotectin level for PDUS synovitis was 2.77; the negative likelihood ratio was 2.29; and these values correctly classified PDUS synovitis in 85 % of patients (Fig. 1). CRP (AUC 0.673) and ESR (AUC 0.731) had a lower discriminatory capacity than calprotectin. An accuracy analysis for DAS28-CRP using PDUS synovitis as the reference variable was also performed. ROC analysis showed a lower accuracy than calprotectin, with an AUC of 0.721 (95 % CI 0.612–0.829, *p* = 0.005) and a cut-off DAS28-CRP value of 1.61 (sensitivity 72 %, specificity 61 %, positive likelihood ratio 1.85, negative likelihood ratio 0.45), and it correctly classified PDUS synovitis in 66.3 % of patients.

Adjusted multivariate regression analysis showed a strong association between calprotectin and PDUS synovitis (OR 4.6, 95 % CI 2.31–9.26, *p* < 0.001) according to the different covariates (combined therapy, reduced dose, use of glucocorticoids, disease duration, and presence of autoantibodies or erosive disease). Backward selection of variables did not substantially modify the association between calprotectin and PDUS synovitis (Table 4). Other variables associated with PDUS synovitis were long-standing disease (OR 1.10, 95 % CI 1.02–1.18, *p* < 0.001) and steroid use (OR 18.1, 95 % CI 2.73–120.2, *p* < 0.005).

**Table 4:** Associations between calprotectin, tumour necrosis factor inhibitor trough serum levels and power Doppler ultrasound synovitis

	Unadjusted β coefficient		Fully adjusted β coefficient	
	OR (95 % CI)	*p* Value	OR (95 % CI)	*p* Value
Calprotectin, μg/ml	3.22 (1.95–5.33)	<0.0001	4.63 (2.31–9.26)	<0.0001
TNFi trough serum levels, μg/ml	0.77 (0.65–0.91)	0.002	0.67 (0.52–0.85)	0.001
Age, years	1.03 (0.99–1.06)	0.164		
Disease duration, years	1.05 (1.00–1.10)	0.067	1.10 (1.02–1.18)	0.016
ESR, mm/h	1.13 (1.05–1.22)	0.001		
Female sex	2.92 (1.18–7.20)	0.020		
Combined therapy, yes or no	1.64 (0.72–3.76)	0.245		
Disease activity according to DAS28-ESR		0.001		
Remission (<2.6)	1			
Low disease activity (2.6–3.2)	7.35 (2.23–24.21)			
Reduced dose, yes or no	0.63 (0.27–1.44)	0.271		
Diagnostic		0.002		
RA	1			
PsA	0.26 (0.11–0.62)			
Steroids, yes or no	2.67 (0.83–8.55)	0.099	18.12 (2.73–120.16)	0.003
Monotherapy, yes or no	0.59 (0.26–1.34)	0.206		
Constant			0.02 (0.003–0.16)	<0.0001

*Abbreviations: DAS28-ESR* 28-joint Disease Activity Score based on erythrocyte sedimentation rate, *ESR* erythrocyte sedimentation rate, *PsA* psoriatic arthritis, *RA* rheumatoid arthritis, *TNFi* tumour necrosis factor inhibitors

### TNFi trough serum levels and power Doppler ultrasound synovitis

RA patients with PDUS synovitis had significantly lower ETN, ADA and IFX trough serum levels. PsA patients with PDUS synovitis had lower trough serum levels of ETN and ADA but not IFX (Table 2). ADA and ETN, but not IFX, trough serum levels inversely correlated with PDUS and MSUS global scores. Similar results were obtained for UdAS (Table 5).

**Table 5:** Correlation between musculoskeletal ultrasound scores and tumour necrosis factor inhibitor trough serum levels

	Total (*n* = 92)	RA (*n* = 42)	PsA (*n* = 50)
Adalimumab			
Doppler score	−0.842^a^	−0.898^a^	−0.803^a^
SH score	−0.329	−0.447	−0.107
Global score	−0.547^b^	−0.639^b^	−0.423^b^
Etanercept			
Doppler score	−0.649^a^	−0.543^b^	−0.791^a^
SH score	−0.024	−0.169	−0.052
Global score	−0.504^b^	−0.251	−0.362
Infliximab			
Doppler score	−0.178	−0.530	−0.076
SH score	−0.052	−0.667	−0.122
Global score	−0.132	−0.870^b^	−0.060

*PsA* psoriatic arthritis, *RA* rheumatoid arthritis, *SH* synovial hypertrophy

Data presented are Spearman’s ρ correlations

^a^ *p* < 0.001

^b^ *p* < 0.05

Although adjusted multivariate regression analysis showed that lower TNFi trough serum levels were significantly associated with PDUS synovitis (OR 0.67, 95 % CI 0.52–0.85, *p* < 0.001), TNFi trough serum levels had low accuracy (AUC <0.5).

### Calprotectin and TNFi trough serum levels

Calprotectin inversely correlated with ADA (ρ = −0.461, *p* = 0.008) and ETN (ρ = −0.436, *p* = 0.005) trough serum levels, although non-significant correlations were found for IFX.

## Discussion

The results of this study show a significant proportion of patients with RA and PsA in remission or with low disease activity who were treated with TNFi had PDUS synovitis, together with significantly higher levels of serum calprotectin, which was more accurate than acute phase reactants (APRs) in identifying PDUS synovitis. Patients with PDUS synovitis had significantly lower TNFi serum levels, and there was an inverse correlation between TNFi serum levels and calprotectin in the two diseases. Therefore, calprotectin and TNFi serum levels may be considered as sensitive biomarkers of synovial inflammation in RA and PsA patients in remission or with low disease activity being treated with TNFi.

Developing standardized instruments to detect synovial inflammation in RA and PsA patients in remission or with low disease activity is a clinical challenge. MSUS, a non-invasive, relatively inexpensive, dynamic technique that allows real-time examination and more sensitive detection of joint inflammation than clinical examination [7], is sufficiently sensitive to measure changes in disease activity, thereby permitting the monitoring of therapeutic responses in patients receiving biological therapy [40].

An increasing amount of data supports the emerging role and clinical utility of imaging techniques such as MSUS in evaluating patients with inflammatory arthritis in clinical remission or with low disease activity. Clinical remission is known not to be equivalent to the absence of imaging-detected synovitis in RA. Only one-third of patients classified as being in clinical remission had a total absence of painful, swollen or tender joints, regardless of the criteria used, and most patients had ongoing active synovitis on the basis of MSUS [41], which may explain the continuing structural progression in some patients despite apparent clinical remission [42].

US-based findings in RA patients in remission should be interpreted with caution. Patients with grade 1 SH, especially those without PD, could be considered to have non-pathologic disease and may be present in the healthy population [43]. In RA patients, SH could suggest the chronic anatomical changes of earlier synovitis rather than current synovial inflammation [44]. In contrast, the presence of PD has repeatedly demonstrated greater sensitivity in detecting active synovitis [11, 12, 15, 45]. In our cohort, 64 % of RA patients and 32 % of PsA patients had PDUS synovitis, although only 24 % of RA patients and 10 % of PsA patients met the more stringent criteria of UdAS. We previously demonstrated that UdAS captured only patients with potentially clinically relevant symptoms and correlated with increased serum levels of angiogenic biomarkers [37].

Subjective clinical symptoms, joint examination and laboratory measurements of APR are not sufficiently sensitive to exclude ongoing inflammation in patients with low disease activity levels. TNFi improves the clinical signs of RA while reducing serum levels of CRP and ESR [46]. The reduction in APRs may be due in part to the direct systemic effect of TNFi on hepatic synthesis of CRP and other APRs, such as fibrinogen, rather than to true improvements in local joint synovitis. In contrast, calprotectin is released by activated phagocytes such as synovial monocytes and granulocytes [18], and its levels reflect local ongoing inflammation rather than a systemic inflammatory response. We recently demonstrated that calprotectin better stratifies disease activity than CRP or ESR in RA patients receiving tocilizumab, a biological agent with a dramatic impact on APRs due to its blockage of interleukin 6 [38]. We also found significant associations between calprotectin and disease status in RA and PsA patients treated with TNFi, with calprotectin levels stratifying disease activity more accurately than CRP and ESR in patients with low levels of inflammation [47].

Calprotectin may be useful in certain groups of patients seen in daily clinical practice, such as patients with inflammatory arthralgia, in order to permit an early diagnosis, or patients with recent-onset RA, in whom CRP and ESR levels may be normal. Clinical assessment of RA patients with comorbidities such as fibromyalgia or osteoarthritis may be challenging; in these patients, calprotectin could help rule out inflammatory activity and elucidate the source of pain, guiding therapeutic decisions (e.g., the initiation or tapering of biological treatment). However, at present, according to our results, one of the most interesting clinical applications may be its use in patients in remission or with low disease activity. It is known that the widely used definition of remission based on DAS28 better represents minimal disease activity than remission. Therefore, calprotectin could rule out ongoing inflammation in these patients and prevent residual activity being undertreated, with the associated therapeutic and prognostic implications.

In only two reports, both about patients with active RA, have authors analysed the association between calprotectin and MSUS. The first included 20 RA patients starting treatment with ADA [20], and the other comprised 37 RA patients taking csDMARDs [48]. Both studies showed significant associations between calprotectin and MSUS, and the second study suggested that calprotectin might be superior to APRs in monitoring US synovitis. To the best of our knowledge, our present study is the first to include PsA patients and to be focused on patients in remission or with low disease activity receiving biological therapy. Our results show that PsA patients had a lower rate of PDUS synovitis than RA patients did. Moreover, as observed in RA patients, PsA patients with PDUS synovitis had significantly higher calprotectin levels. Calprotectin correlated better and more consistently with MSUS than APRs, and we found significant correlations between calprotectin and PD, SH and US global scores. The US global score combines inflammatory (power Doppler) and structural findings (synovial hypertrophy) in an attempt to capture the whole state of the joint, although previous studies have shown that only the PD score was associated with radiographic progression [49]. In light of the strong correlation in our present study, it seems logical that calprotectin and PDUS synovitis both identify local active synovitis in patients with low levels of disease activity.

This is particularly interesting, considering recent evidence suggesting that TNF blockade depends on the trough serum levels achieved. It is reported that patients with a good response to TNFi therapy have higher drug trough serum levels [24, 25]. Our results show that patients with PDUS synovitis had lower trough serum levels. TNFi trough serum levels inversely correlated with PD in patients receiving ADA and ETN (all *r* coefficients >0.50), although they were less accurate than calprotectin in detecting PDUS synovitis. In a previous study, researchers detected no significant influence of anti-TNF pharmacokinetics on the SH and PD scores in RA patients, although TNFi trough serum levels were not measured in that study [50]. Therefore, our study is the first to be focused on the relationship between TNFi trough serum levels and MSUS assessment in RA and PsA patients.

Our study had some limitations. First, this was a cross-sectional study in patients with long-standing RA and PsA who were receiving prolonged TNFi therapy, and therefore the value of calprotectin, TNFi trough serum levels and PDUS synovitis as prognostic factors for flares or in monitoring therapeutic responses to biological therapy was not evaluated. Second, we used DAS28 ≤ 3.2 as the inclusion criterion. This allowed residual tender and/or swollen joints in patients classified as being in remission and did not include the feet. However, it is the most widely used composite articular index, and therefore our results would be clinically applicable. Although the DAS28 was developed to assess disease activity in RA, studies support its use in PsA patients receiving biological therapy [30]. Third, concomitant treatment with csDMARDs, steroids and non-steroidal anti-inflammatory drugs was not standardized, owing to the observational nature of the study. Finally, US assessment was performed by only one rheumatologist, and therefore inter-observer reliability could not be calculated. This may be a weakness of the study because of the known significant variability between observers in the US assessment of synovitis [51].

## Conclusions

PsA patients in remission or with low disease activity had a lower rate of PDUS synovitis than RA patients. Patients with PDUS synovitis had higher calprotectin levels and lower TNFi trough serum levels, reflecting local, ongoing synovial inflammation. Calprotectin was more accurate than APRs in detecting PDUS synovitis in these patients. TNFi trough serum levels inversely correlated with the PD score and calprotectin levels. Calprotectin and drug trough serum levels may help clinicians identify US active synovitis in RA and PsA patients in clinical remission or with low disease activity treated with TNFi.

## Abbreviations

ADA, adalimumab; AIC, Akaike information criterion; APR, acute phase reactant; CDAI, Clinical Disease Activity Index; CRP, C-reactive protein; csDMARD, conventional synthetic disease-modifying anti-rheumatic drug; DAS28-ESR, 28-joint Disease Activity Score based on erythrocyte sedimentation rate; ELISA, enzyme-linked immunosorbent assay; ESR, erythrocyte sedimentation rate; ETN, etanercept; IFX, infliximab; MRP, myeloid-related protein; MSUS, musculoskeletal ultrasound; OMERACT, Outcome Measures in Rheumatoid Arthritis Clinical Trials; PD, power Doppler; PDUS, power Doppler ultrasound; PsA, psoriatic arthritis; RA, rheumatoid arthritis; SDAI, Simple Disease Activity Index; SH, synovial hypertrophy; SJC, swollen joint count; TJC, tender joint count; TNFi, tumour necrosis factor inhibitors; UdAS, ultrasound-defined active synovitis; US, ultrasound; NV, normal value
